# Effects of a polypill on circulating levels of resistin and visfatin in men with non-alcoholic fatty liver disease: A five-year clinical trial

**DOI:** 10.1371/journal.pone.0331121

**Published:** 2025-10-08

**Authors:** Mahdieh Nazari-Robati, Tania Dehesh, Beydolah Shahouzehi, Gholamreza Roshandel, Hossein Poustchi, Solaleh Emamgholipour

**Affiliations:** 1 Neuroscience Research Center, Institute of Neuropharmacology, Kerman University of Medical Sciences, Kerman, Iran; 2 Department of Clinical Biochemistry, Faculty of Medicine, Kerman University of Medical Sciences, Kerman, Iran; 3 Modeling in Health Research Center, Institute for Futures Studies in Health, Kerman University of Medical Sciences, Kerman, Iran; 4 Cardiovascular Research Center, Institute of Basic and Clinical Physiology Sciences, Kerman University of Medical Sciences, Kerman, Iran; 5 Golestan Research Center of Gastroenterology and Hepatology, Golestan University of Medical Sciences, Gorgan, Iran; 6 Liver and Pancreaticobiliary Disease Research Center, Digestive Diseases Research Institute, Tehran University of Medical Sciences, Tehran, Iran; 7 Department of Clinical Biochemistry, Faculty of Medicine, Tehran University of Medical Sciences, Tehran, Iran; University of Montenegro-Faculty of Medicine, MONTENEGRO

## Abstract

Non-alcoholic fatty liver disease (NAFLD) is the most prevalent liver disease globally, characterized by insulin resistance, hypertension, and obesity. Adipokines such as resistin and visfatin play significant roles in glucose homeostasis and lipid metabolism. Polypills are utilized to improve cardiovascular disease (CVD) risk factors. The present observational study was nested within the PolyIran-Liver randomized controlled trial, which primarily assessed clinical outcomes in NAFLD patients. This study aimed to evaluate the effects of prolonged polypill consumption (five years) on circulating levels of resistin and visfatin as secondary outcomes in men with NAFLD. Participants from the PolyIran-Liver trial were included, comprising 41 patients in the control group and 40 patients in the polypill group, all of whom were men. The polypill regimen included aspirin, hydrochlorothiazide, atorvastatin, and valsartan. Treatment with the polypill resulted in a significant reduction in visfatin levels (2.27 ± 0.83 ng/ml vs. 2.10 ± 0.71 ng/ml, AdjP = 0.041), but no significant changes in resistin levels were observed within the polypill group (19.54 ± 4.11 ng/ml vs. 19.11 ± 3.08 ng/ml, AdjP = 0.396). The reduction in visfatin levels from baseline was significantly associated with changes in resistin levels and fasting blood glucose (FBG) (P < 0.05). Additionally, polypill intervention improved alanine aminotransferase (ALT) levels, lipid profiles, and systolic blood pressure in patients with NAFLD (P < 0.05). Our findings suggest that daily intake of the polypill can lead to significant reductions in visfatin levels and improvements in metabolic parameters in men with NAFLD. Further studies are needed to evaluate the long-term implications of polypill consumption in managing NAFLD through targeting adipokines.

## Introduction

Non-alcoholic fatty liver disease (NAFLD) is the most common cause of liver dysfunction worldwide, affecting approximately 25% of the global population [1]. The prevalence of NAFLD increases concurrently with the ongoing epidemics of obesity and type 2 diabetes mellitus (T2DM). NAFLD is also associated with extra-hepatic complications, such as cardiovascular diseases (CVDs), hypertension, and chronic kidney disease (CKD), which are major contributors to mortality among NAFLD patients [2]. The development of NAFLD is linked to dyslipidemia characterized by fat accumulation in hepatocytes, which can progress to an inflammatory stage termed non-alcoholic steatohepatitis (NASH), fibrosis, and ultimately hepatocellular carcinoma [3]. Inflammatory adipokines, secreted by adipose tissue, have emerged as important factors involved in the pathogenesis of NAFLD and its progression to NASH and fibrosis, making them potential therapeutic targets for NAFLD [4].

Resistin belongs to a family of cysteine-rich peptides, which is predominantly produced by adipose tissue and inflammatory cells. Resistin exerts various effects, including the regulation of serum glucose levels and lipid metabolism, as well as the induction of inflammation [4]. Elevated levels of resistin have been shown to induce insulin resistance [5]. Moreover, the increase in circulating resistin in obese individuals and those with T2DM suggests that resistin serves as a pivotal link between obesity and diabetes [6]. Resistin is also associated with hepatic fibrosis through its pro-inflammatory action. Circulating resistin levels have been found to increase in patients with NAFLD and NASH, which were positively related to the severity of steatosis, inflammation, and fibrosis [7].

Visfatin, known as nicotinamide phosphoribosyltransferase (NAMPT), is an adipokine produced by various tissues, including adipose tissue, hepatocytes, and leukocytes [8]. Visfatin has been recognized as an important factor in regulating beta-cell function, glucose homeostasis, and lipid metabolism [9]. It also acts as a proinflammatory adipokine and induces the expression of cytokines which are associated with the development of insulin resistance [10]. Visfatin has been confirmed to interfere with insulin signaling pathways, resulting in a reduction in glucose uptake by adipocytes and an increase in adipogenesis [11]. Elevated levels of visfatin have been reported in patients with obesity, T2DM, and metabolic syndrome [12]. The role of visfatin in NAFLD has been extensively studied but the findings have been contradictory. Several studies have reported augmented levels of circulating visfatin in subjects with NAFLD, which were associated with the severity of hepatic steatosis and fibrosis [13–15].

The polypill is a multi-component tablet composed of a statin, an antiplatelet agent, and antihypertensive drugs. Initially developed to reduce the incidence of cardiovascular events in patients with a history of CVD, it has also been administered to individuals over the age of 55 [16]. Evidence suggests that polypills are particularly effective in managing hypertension, dyslipidemia, and ischemic cardiomyopathy [17]. Although CVD is the leading cause of mortality among patients with NAFLD, data on the effects of polypill therapy in this population remain limited [18]. Recently, findings from a long-term clinical trial demonstrated that the polypill is both safe and effective in reducing cardiovascular events in patients with NAFLD, and may also contribute to the reduction of liver enzyme levels [19]. Despite the established associations between NAFLD and adipokines, particularly resistin and visfatin, evidence regarding the impact of pharmacological interventions such as polypills on these biomarkers in patients with NAFLD is scarce. The present study was conducted as an observational study nested within a previously implemented randomized controlled trial (the PolyIran-Liver study), which primarily aimed to assess clinical outcomes including liver-related parameters and metabolic markers. The current study aimed to evaluate circulating levels of resistin and visfatin as secondary outcomes to investigate the potential mechanistic effects of prolonged polypill consumption in patients with NAFLD.

## Materials and methods

### Study design

This study utilized samples from a previously conducted clinical trial, the PolyIran-Liver study, an open-label randomized controlled trial nested within the Golestan cohort study (ID: NCT01245608). Within the framework of this randomized controlled trial, we measured circulating levels of resistin and visfatin among the same participants enrolled in the main trial. The selection process was conducted using a computer-generated random number sequence to ensure unbiased allocation from the main randomized clinical trial.

### Participants

Participation in the PolyIran-Liver study was voluntary. All participants gave written informed consent for the study. Patients included in the PolyIran-Liver research were those who had been newly diagnosed with NAFLD and were aged over 50 years. The diagnosis of NAFLD was performed by ultrasonography. Participants with alcohol use, active hepatitis, and clinical cirrhosis were excluded from the study. The selection process involved random assignment into two groups: an intervention group receiving a polypill and a control group. The randomization was conducted using a computer-generated random number sequence to ensure unbiased allocation. Participants in the control group received lifestyle modification advice, including physical activity and dietary change. These recommendations were based on established guidelines for NAFLD management. Patients in the polypill group were asked to take one pill per day in addition to lifestyle modification [20]. The polypill contained 80 mg of aspirin, 12.5 mg of hydrochlorothiazide, 20 mg of atorvastatin, and 40 mg of valsartan. All participants were followed for five years to assess the long-term effects of the intervention. Participants in both groups were visited every 6 months. Patients in the intervention group had two more visits at 1 and 2 months after starting their treatment to check for important adverse events. In each follow-up visit, anthropometric and blood pressure measurements were performed. Adherence to polypill was calculated at each visit and the entire duration of the study. The adherence was calculated by dividing the number of pills used by the participant by the number of pills supplied. The design, inclusion, and exclusion criteria have been detailed in previous studies [19,21].

Original samples and data were accessed on 20 October 2023. Authors had no access to information that could identify individual participants during or after sample collection. From the total cohort of the PolyIran-Liver study, we selected 40 patients from the polypill group and 41 patients from the control group for our analysis. All patients were men. To ensure unbiased group assignment, participants were randomized using a computer-generated random number sequence. Regarding the inclusion of only male participants, this decision was based on the study’s focus on evaluating specific metabolic parameters and adipokines in a more homogeneous sample. Gender differences in lipid metabolism and the expression of adipokines may introduce variability that could confound the study outcomes. Therefore, to minimize confounding factors and enhance the homogeneity of the study population, only male participants were included. In addition, according to our previous study, high adherence to polypill was related to male sex [19]. Therefore, we selected male subjects from the polypill group randomly. Patients from the control group were also men and matched on age and NAFLD stage. The total fatty liver score for all men with NAFLD was 1–3 (mild fatty liver). The level of NAFLD was the same across all participants in both the control and intervention groups. The study protocol was approved by the local ethics committee of Kerman University of Medical Sciences (IR.KMU.REC.1401.290) and conducted in accordance with the Helsinki Declaration.

### Laboratory measurements

The levels of resistin and visfatin in serum samples were measured using enzyme-linked immunosorbent assay (ELISA) kits (Zellbio, Germany). The assays were performed according to the manufacturer’s instructions, ensuring that all samples were analyzed in duplicate to enhance the accuracy of the assay. Quality control measurements included running known standards alongside patient samples to validate assay performance. Variations between duplicate samples were less than 10%. The mean concentrations were calculated and reported.

### Biochemical and clinical data collection

In addition to measuring resistin and visfatin levels, we collected comprehensive biochemical and clinical data from participants throughout the study. These data were obtained from routine clinical evaluations conducted as part of the PolyIran-Liver study which encompassed demographic information (age), anthropometric measurements (weight, height, waist circumference), hemodynamic parameters (blood pressure), and biochemical tests (liver enzymes, lipid profiles and blood glucose levels). Liver stiffness measurement (LSM) was also performed using the FibroScan machine (Echosens, France) via the M or XL probes as appropriate. The optimal cut-off LSM values were <7 kPa for no or mild fibrosis (F0 to F1), 7–10 kPa for moderate fibrosis (F2), 10–14 kPa for severe fibrosis (F3) and ≥14 kPa for cirrhosis (F4).

### Statistical analysis

Data analysis was performed using the SPSS statistical software package, version 21.0 (SPSS Inc., Chicago, IL, USA). Quantitative variables were reported as means with standard deviations (SD), while categorical variables were expressed as frequencies and percentages. Baseline comparisons between the two groups were conducted using the independent t-test for continuous variables and the Chi-square (χ²) test for categorical variables. To assess differences in changes from baseline to the end of the intervention, analysis of covariance (ANCOVA) was employed. Prior to applying ANCOVA, the assumptions of normality and homogeneity of variances were evaluated using the Kolmogorov–Smirnov test and Levene’s test, respectively. Additionally, the homogeneity of regression slopes (i.e., absence of interaction between group assignment and baseline covariate) was assessed. All ANCOVA models were adjusted for baseline variables that differed significantly between the groups. Furthermore, stepwise multiple regression analysis was conducted to identify independent predictors of change in visfatin levels. A p-value of less than 0.05 was considered statistically significant.

## Results

### Participant demographics

Details of the enrolment process and inclusion criteria were published previously [21]. All variables had normal distribution and parametric tests were used to analyze data. The baseline demographics and laboratory characteristics of 81 patients with NAFLD have been shown in Table 1. All participants were men with the mean ages of 59.05 ± 5.19 years in the control group and 60.13 ± 3.98 years in the intervention group.

**Table 1:** Baseline demographics and laboratory characteristics of men with NAFLD.

Variable	Control N = 41	Polypill N = 40	P value
Age (year)	59.05 ± 5.19	60.13 ± 3.98	0.301
Smoking			
Yes, N (%)	5 (12.2)	2 (5.0)	0.433
No, N (%)	36 (87.8)	38 (95.0)	
Anthropometrics			
Height (cm)	168.62 ± 6.58	166.87 ± 6.12	0.220
Weight (Kg)	83.49 ± 11.79	85.27 ± 10.68	0.477
BMI (Kg/m^2^)	29.34 ± 3.68	30.64 ± 3.69	0.094
Waist circumference (cm)	105.36 ± 10.64	106.50 ± 9.08	0.528
Blood pressure			
Systolic (mmHg)	131.18 ± 20.20	135.18 ± 18.44	0.473
Diastolic (mmHg)	81.96 ± 8.88	84.13 ± 10.08	0.340
FBG (mg/dl)	106.78 ± 26.43	106.68 ± 24.60	0.984
Lipids, fasting			
Triglycerides (mg/dl)	161.37 ± 75.52	154.35 ± 81.79	0.615
Total cholesterol (mg/dl)	195.89 ± 36.49	223.98 ± 33.01	<0.001
HDL-c (mg/dl)	49.91 ± 12.77	56.35 ± 12.40	0.024
LDL-c (mg/dl)	115.38 ± 31.56	136.75 ± 25.71	0.001
LDL-c/HDL-c	2.36 ± 0.68	2.50 ± 0.53	0.280
Total cholesterol/HDL-c	4.05 ± 0.83	4.12 ± 0.89	0.719
Liver enzymes and stiffness			
AST (U/L)	25.06 ± 12.19	23.86 ± 8.97	0.657
ALT (U/L)	34.27 ± 24.94	28.72 ± 10.98	0.217
ALP (U/L)	234.78 ± 86.48	235.73 ± 46.53	0.859
LSM (kPa)	6.15 ± 2.18	6.38 ± 2.08	0.541
Adipokines			
Resistin (ng/ml)	22.05 ± 4.95	19.54 ± 4.11	<0.001
Visfatin (ng/ml)	2.83 ± 1.28	2.27 ± 0.83	0.006
NAFLD stage	1.46 ± 0.10	1.37 ± 0.10	0.527

Abbreviations: NAFLD, non-alcoholic fatty liver disease; BMI, body mass index; FBG, fasting blood glucose; HDL-c, high-density lipoprotein cholesterol; LDL-c, low-density lipoprotein cholesterol; AST, aspartate aminotransferase; ALT, alanine aminotransferase; ALP, alkaline phosphatase; LSM, liver stiffness measurement.

P values were obtained from independent t-test. Data are expressed as mean±SD for quantitative variables and frequency (percentage) for categorical variables.

### Baseline characteristics

Baseline values for weight, waist circumference, height, body mass index (BMI), smoking status, blood pressure, triglycerides, and fasting blood glucose (FBG) did not differ significantly between the control and polypill groups (Table 1). Moreover, there were no statistically significant differences between the two groups in baseline levels of serum markers of liver injury, including alanine aminotransferase (ALT), aspartate aminotransferase (AST), alkaline phosphatase (ALP), or LSM. However, significant differences were observed in baseline levels of total cholesterol, high-density lipoprotein cholesterol (HDL-c), and low-density lipoprotein cholesterol (LDL-c), as well as in serum concentrations of resistin and visfatin between the control and intervention groups.

### Effects of polypill intervention

Table 2 presents the mean values of parameters measured at baseline and after five years of polypill treatment. The results showed no significant changes or between-group differences in BMI. After adjustment for confounding factors, the difference in BMI between the two groups remained non-significant (P > 0.05). In contrast, the mean waist circumference decreased significantly in the control group after five years (P < 0.001), with a greater reduction compared to the polypill group (P = 0.014). Regarding blood pressure, the within-group analysis revealed significant reductions in both systolic and diastolic blood pressure in both groups. However, between-group differences were not significant (P > 0.05). After adjusting for baseline covariates, a significant reduction in systolic blood pressure was observed in the control group compared to the polypill group (P = 0.044).

**Table 2:** A comparison of anthropometric, laboratory, and clinical measures, along with visfatin and resistin levels, before and after intervention in men with NAFLD.

Variable	Control			Polypill			P value^2^	Adjusted P value^3^
	Baseline	After 5 years	P value^1^	Baseline	After 5 years	P value^1^		
Smoking								
Yes, N (%)	5 (12.2)	3 (7.3)	0.457	2 (5.0)	1 (2.5)	0.556	0.571	0.621
No, N (%)	36 (87.8)	38 (92.7)		38 (95.0)	39 (97.5)			
BMI (Kg/m^2^)	29.34 ± 3.68	29.69 ± 5.85	0.584	30.64 ± 3.69	30.59 ± 4.03	0.638	0.486	0.865
Waist circumference (cm)	105.36 ± 10.64	99.68 ± 13.46	<0.001	106.50 ± 9.08	106.53 ± 9.81	0.628	0.001	0.014
Blood pressure								
Systolic (mmHg)	131.18 ± 20.20	119.47 ± 24.60	0.007	135.18 ± 18.44	119.25 ± 19.03	<0.001	0.719	0.044
Diastolic (mmHg)	81.96 ± 8.88	70.78 ± 12.73	<0.001	84.13 ± 10.08	69.87 ± 8.06	<0.001	0.538	0.176
FBG (mg/dl)	106.78 ± 26.43	119.70 ± 47.20	0.073	106.68 ± 24.60	112.87 ± 32.86	0.193	0.498	0.600
Lipids, fasting								
Triglycerides (mg/dl)	161.37 ± 75.52	154.11 ± 74.68	0.504	154.35 ± 81.79	125.74 ± 62.42	0.01	0.261	0.027
Total cholesterol (mg/dl)	195.89 ± 36.49	186.39 ± 41.77	0.169	223.98 ± 33.01	158.79 ± 39.19	<0.001	<0.001	<0.001
HDL-c (mg/dl)	49.91 ± 12.77	53.78 ± 13.01	0.001	56.35 ± 12.40	53.36 ± 12.17	0.006	<0.001	<0.001
LDL-c (mg/dl)	115.38 ± 31.56	101.79 ± 31.80	0.03	136.75 ± 25.71	80.28 ± 31.85	<0.001	<0.001	<0.001
LDL-c/HDL-c	2.36 ± 0.68	1.94 ± 0.55	<0.001	2.50 ± 0.53	1.53 ± 0.56	<0.001	<0.001	<0.001
Total cholesterol/HDL-c	4.05 ± 0.83	3.53 ± 0.71	<0.001	4.12 ± 0.89	3.05 ± 0.74	<0.001	<0.001	<0.001
Liver enzymes and stiffness								
AST (U/L)	25.06 ± 12.19	19.68 ± 7.51	0.005	23.86 ± 8.97	20.09 ± 7.86	0.008	0.567	0.504
ALT (U/L)	34.27 ± 24.94	20.47 ± 9.25	<0.00	28.72 ± 10.98	24.79 ± 8.23	0.074	0.001	0.007
ALP (U/L)	234.78 ± 86.48	222.05 ± 73.62	0.083	235.73 ± 46.53	215.87 ± 40.39	<0.001	0.386	0.613
LSM (kPa)	6.15 ± 2.18	4.86 ± 1.59	0.001	6.38 ± 2.08	4.62 ± 1.16	<0.00	0.461	0.312
Adipokines								
Resistin (ng/ml)	22.05 ± 4.95	19.52 ± 2.31	0.003	19.54 ± 4.11	19.11 ± 3.08	0.594	0.653	0.396
Visfatin (ng/ml)	2.83 ± 1.28	2.64 ± 1.25	0.398	2.27 ± 0.83	2.10 ± 0.71	0.309	0.091	0.041

Abbreviations: NAFLD, non-alcoholic fatty liver disease; BMI, body mass index; FBG, fasting blood glucose; HDL-c, high-density lipoprotein cholesterol; LDL-c, low-density lipoprotein cholesterol; AST, aspartate aminotransferase; ALT, alanine aminotransferase; ALP, alkaline phosphatase; LSM, liver stiffness measurement.

1. P values were obtained from paired t-test. 2. P values were obtained from unadjusted analysis of covariance (ANCOVA). 3. P values were obtained from ANCOVA adjusted for variables that differed at baseline between control and polypill groups. Data are expressed as mean±SD for quantitative variables and frequency (percentage) for categorical variables.

Serum FBG levels showed a numerical increase in both groups, though this was not statistically significant. The FBG levels were slightly higher in the control group than in the polypill group, but the difference between groups was not meaningful (P > 0.05).

Analysis of the serum lipid profile revealed reductions in triglycerides and total cholesterol in both groups after five years of treatment. However, the reduction in these parameters was significantly greater in the polypill group compared to the control group. The control group showed significant improvements in HDL-c and LDL-c levels, as well as in the LDL-c/HDL-c and total cholesterol/HDL-c ratios. While similar improvements were observed in the polypill group, ANCOVA analysis indicated that the improvements in lipoprotein profiles were significantly greater in the polypill group (P < 0.05).

Within-group analysis (paired t-test) demonstrated improvements in liver enzyme levels in both groups, with significant reductions in AST and ALT in the control group and in AST and ALP in the intervention group. However, the between-group comparison revealed a significantly greater reduction in ALT in the control group compared to the polypill group (P = 0.007). Consistent with improvements in liver enzymes, LSM was reduced in both groups, but the difference between the control and intervention groups was not statistically significant, either with or without adjusting for potential confounding factors (P > 0.05).

As for adipokines, a significant reduction in resistin levels was observed in the control group, while visfatin levels did not show significant changes. In the polypill group, neither resistin nor visfatin levels changed significantly. ANCOVA analysis revealed no significant differences in changes in resistin and visfatin between the two groups after five years. However, after adjusting for baseline covariates, a significant difference in visfatin levels was detected between the control and intervention groups (P = 0.041). The intervention group demonstrated lower levels of visfatin after adjustment for baseline covariates.

**Fig 1:**
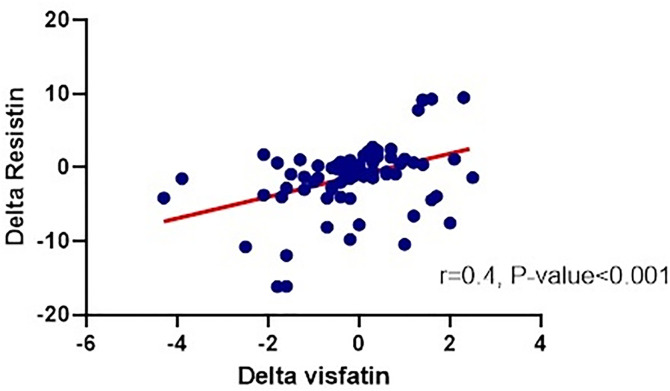
Correlation between visfatin and resistin levels within the polypill group.

For each variable, post-treatment values were subtracted from baseline values to calculate the delta, which was used to express the change in the variable. To assess the associations between changes in visfatin and other variables, multiple regression analysis was performed using delta values for each group. The results indicated that in the control group, the reduction in visfatin was significantly associated with a decrease in LSM (P = 0.035) (Table 3). In contrast, a significant association was observed between the reduction in visfatin and the decrease in resistin in the polypill group (r = 0.4, P < 0.001) (Fig 1). Furthermore, in the polypill group, the reduction in visfatin was significantly associated with an increase in FBG levels (P = 0.023).

**Table 3:** Multiple regression analysis with baseline visfatin in the model for control and polypill groups.

	β	95% confidence interval	t	P value
**Control group**				
Baseline visfatin	0.410	0.174, 0.646	3.462	0.001
ΔLSM	0.122	0.009, 0.235	2.147	0.035
**Polypill group**				
Baseline visfatin	0.390	0.126, 0.654	3.012	0.005
ΔResistin	0.183	0.143, 0.224	9.190	<0.001
ΔFBG	−0.005	−0.010, −0.001	−2.390	0.023

Abbreviations: β, regression coefficient; LSM, liver stiffness measure; Δ, change in; FBG, fasting blood glucose.

## Discussion

The primary objective of this study was to investigate the effects of a polypill on circulating levels of resistin and visfatin in men with NAFLD. Our results demonstrated that polypill consumption significantly reduced the level of visfatin in these patients; however, it did not produce a statistically significant effect on resistin levels. Notably, the reduction in visfatin from baseline was associated with changes in both resistin and FBG. Furthermore, improvements were observed in ALT levels, lipid profile, and systolic blood pressure in the polypill group.

The polypill contains a combination of widely used medications aimed at controlling CVD and related pathologies [22]. CVD is prevalent among individuals with NAFLD and represents the primary cause of death in NAFLD patients [18]. In line with our findings, a recent clinical trial indicated that the polypill is both safe and effective against CV events and reduces the levels of liver enzymes in subjects with NAFLD [19]. This suggests that the polypill may have beneficial effects beyond managing cardiovascular risk factors, potentially influencing metabolic pathways associated with liver health.

Our study contributes to the expanding body of literature highlighting adipokines as key mediators in metabolic disorders. Several studies have indicated elevated levels of resistin and visfatin in patients with NAFLD compared to control groups [23,24]. The findings of the current study indicated that the polypill alleviates the level of visfatin in men with NAFLD, whereas no significant changes were observed in resistin levels following a 60-month treatment with the polypill. A possible explanation for the aforementioned finding could be attributed to the factors that lie beyond the direct scope of the polypill’s actions. These factors might include individual differences in genetic predisposition, variations in baseline inflammatory or metabolic states, or environmental influences such as diet, physical activity, and stress levels [25–27]. Additionally, other biochemical pathways or regulatory mechanisms not directly targeted by the components of the polypill could play a role in modulating visfatin and resistin levels. For instance, changes in hormonal regulation, including insulin and glucocorticoid signaling pathways may profoundly influence the secretion and action of these adipokines [28]. Moreover, microRNAs that modulate gene expression at post-transcriptional levels are implicated in the regulation of adipokines under various physiological and pathological conditions [29]. DNA modifications, such as DNA methylation and histone modifications can also affect gene expression and contribute to long-term alterations in adipokine regulation [30]. Hence, it seems that observed findings may result from an interplay of broader physiological processes rather than the polypill alone. However, a larger sample size can provide better reliability and robustness to the findings. In contrast, multiple regression analysis revealed a positive association between variations in visfatin and resistin levels within the polypill group indicating the possibility of a shared or interconnected regulatory mechanism. Both visfatin and resistin may be regulated by systemic inflammatory or metabolic states and the changes induced by the polypill in these states could potentially explain the observed correlation. Additionally, this relationship could represent a compensatory or synergistic interaction between visfatin and resistin within adipose tissue biology or in response to metabolic alterations. These findings highlight the complexity of adipocytokine regulation and emphasize the intricate effects of long-term polypill therapy on metabolic biomarkers.

The participants in the control group were advised to adopt lifestyle changes, such as increasing physical activity. Recent guidelines for the treatment of NAFLD primarily focus on weight reduction through lifestyle modifications [31]. Currently, no specific medication has been approved for the treatment of NAFLD. The proposed pharmacological management of NAFLD aims to treat other aspects of the disease, including dyslipidemia and insulin resistance [32]. In this study, NAFLD patients were treated with the polypill containing 20 mg of atorvastatin. This dose is the most frequently prescribed dose of atorvastatin, which improved the lipid profile, in particular LDL-c in these patients. In addition, daily administration of a single polypill led to a significant reduction in fasting levels of circulating lipids compared to the lifestyle intervention after five years. However, the improvement in serum lipid levels was not associated with the reduction in visfatin. Consistent with this finding, several studies have reported that serum levels of visfatin do not correlate with the lipid profile in obese individuals and patients with NAFLD [14,33].

Regarding liver enzymes, our data revealed that the mean values of AST, ALT, and ALP were in the normal range. Both the lifestyle intervention and the polypill therapy resulted in reductions in circulating liver enzyme levels. However, after five years, men in the control group experienced a greater reduction in ALT levels. NAFLD is typically asymptomatic, and liver enzyme levels are often either normal or mildly elevated. Therefore, the diagnosis of NAFLD requires evidence of hepatic steatosis, as confirmed through imaging or histological examination [34]. In this study, the within-group analysis showed that both polypill and lifestyle interventions potentially reduced the level of LSM determined by transient elastography. Furthermore, our analysis revealed that reductions in visfatin were significantly correlated with decreases in LSM within the control group, indicating a potential relationship between visfatin levels and liver stiffness, an important indicator of liver health and fibrosis progression in patients with NAFLD. In contrast, within the polypill group, reductions in visfatin were significantly associated with decreases in resistin levels. The observed association implies that the modulation of visfatin by the polypill could potentially lead to changes in resistin levels, highlighting the need for further research to elucidate the underlying mechanisms driving these relationships.

NAFLD patients in both groups experienced a slightly higher FBG after five years, indicating that none of the interventions, particularly lifestyle modifications, could improve hyperglycemia in these patients. The results also showed a negative association between changes in circulating levels of visfatin and FBG. This finding is consistent with the results of several studies [35,36]. Visfatin has been proposed as an insulin-mimicking adipokine that stimulates glucose uptake and lipogenesis [37]. It is therefore possible that the reduction in visfatin impaired glucose uptake and metabolism, leading to a higher level of FBG. On the other hand, visfatin has well-established roles in promoting inflammation and fibrosis, both of which are key factors in the progression of NAFLD. As a proinflammatory adipokine, visfatin can stimulate the release of cytokines such as TNF-α and IL-6, which contribute to liver inflammation and the development of fibrosis [38,39]. Visfatin promotes fibrogenesis by increasing the expression of transforming growth factor-β (TGF-β) and other fibrogenic factors in the liver [29,40]. The reduction in visfatin may therefore mitigate inflammation and fibrosis, potentially slowing disease progression in NAFLD. However, the observed negative association between visfatin and FBG highlights a complex interplay between visfatin’s effects on glucose metabolism and liver disease. While visfatin reduction may offer advantages in terms of reducing inflammation and fibrosis, these potential benefits must be considered alongside its role in glucose homeostasis. Therefore, further studies are necessary to fully elucidate the dual roles of visfatin in both metabolic regulation and liver pathology. While our study provides valuable insights into the effects of the polypill on adipokine levels in men with NAFLD, several limitations must be acknowledged. First, our sample size was relatively small, which may limit the generalizability of our findings. Additionally, as this research was an observational study nested within a larger trial, we cannot establish causality between polypill treatment and changes in adipokine levels. Future studies should aim to include larger cohorts and consider longitudinal designs to better understand these relationships. Finally, this study was not a placebo-controlled trial, and lifestyle modifications, including physical activity and dietary changes were not evaluated which presents a potential area for future research using more intensive monitoring strategies.

## Conclusion

Taken together, our findings showed that daily intake of the polypill for five years can lead to significant reductions in visfatin levels and improvements in metabolic parameters among men with NAFLD. However, further research is warranted to explore the underlying mechanisms driving these changes and to assess the long-term implications for managing NAFLD through pharmacological interventions targeting adipokines.

## Supporting information

S1 Data Dataset. (XLSX)

## Abbrevations:

ALP: alkaline phosphatase
ALT: alanine aminotransferase
AST: aspartate aminotransferase
BMI: body mass index
CKD: chronic kidney disease
CVD: cardiovascular disease
ELISA: enzyme-linked immunosorbent assay
FBG: fasting blood glucose
HDL-c: high-density lipoprotein cholesterol
LDL-c: low-density lipoprotein cholesterol
LSM: liver stiffness measurement
NAFLD: Non-alcoholic fatty liver disease
NAMPT: nicotinamide phosphoribosyltransferase
NASH: non-alcoholic steatohepatitis
T2DM: type 2 diabetes mellitus
